# Supplementary material for: Ten-eleven translocation 2 interacts with forkhead box O3 and regulates adult neurogenesis
Source: Nat Commun. 2017 Jun 29;8:15903. doi: 10.1038/ncomms15903 (PMC5493768; doi:10.1038/ncomms15903)
Supplement: Supplementary Information — Supplementary Figures. [file ncomms15903-s1.pdf]

Type of file: pdf
Size of file: 0 KB
Title of file for HTML: Supplementary Information
Description: Supplementary Figures.

Type of file: XLSX
Size of file: 0 KB
Title of file for HTML: Supplementary Data 1
Description: Genome-wide transcriptome changes in wildtype and *Tet2-/-* aNSCs

Type of file: XLSX
Size of file: 0 KB
Title of file for HTML: Supplementary Data 2
Description: Merged Foxo3a ChIP-seq peaks from wildtype and *Tet2-/-* aNSCs.

Type of file: XLSX
Size of file: 0 KB
Title of file for HTML: Supplementary Data 3
Description: Merged 5hmC peaks from wildtype and *Tet2-/-* aNSCs.

Type of file: XLSX
Size of file: 0 KB
Title of file for HTML: Supplementary Data 4
Description: Upregulated genes in the absence of Tet2 that are involved in the cell cycle and nervous system development.

Type of file: XLSX
Size of file: 0 KB
Title of file for HTML: Supplementary Data 5
Description: Sequencing quality report of 5hmC-seq, Foxo3a ChIP-seq and RNA-seq.

Type of file: pdf
Size of file: 0 KB
Title of file for HTML: Peer Review File
Description:

Supplementary Figures

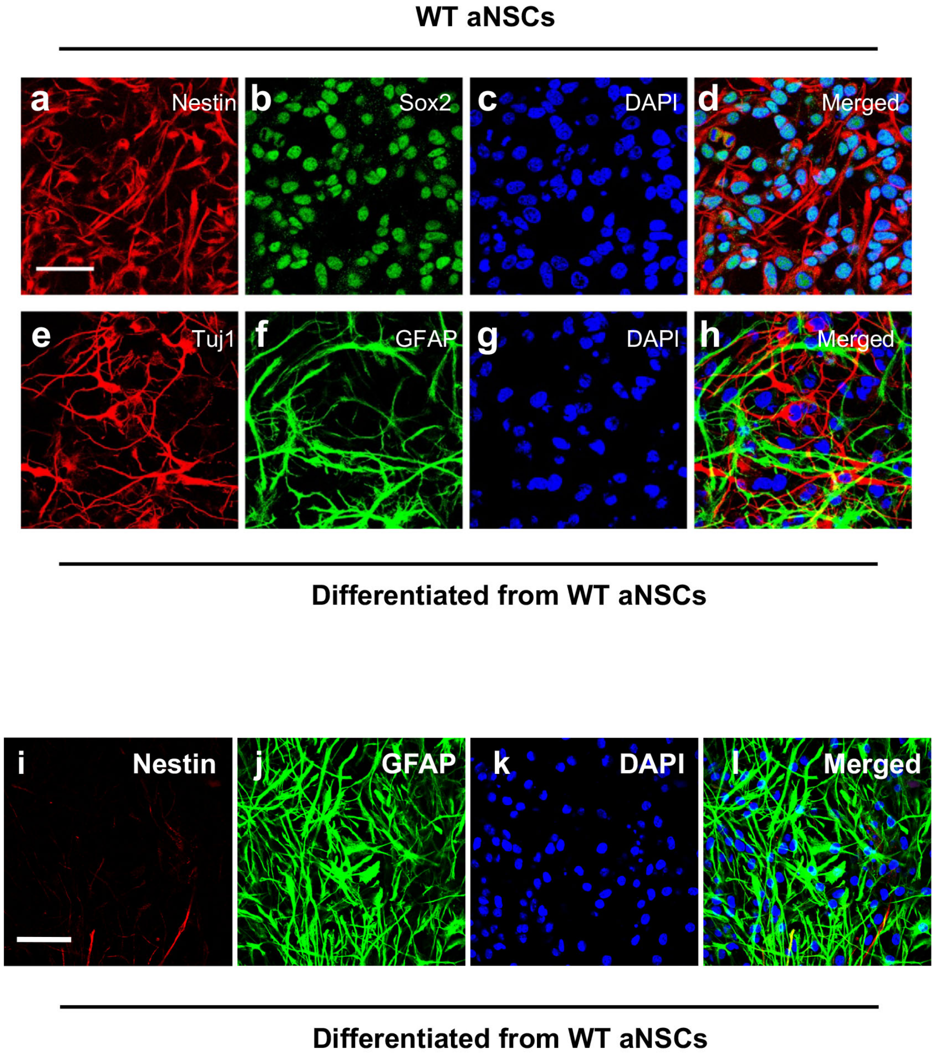

Supplementary Figure 1. Proliferation and differentiation assays of cultured aNSCs *in vitro*.
(a-d) Representative immunostaining (IF) images of Nestin^{+} aNSCs with (a) aNSC marker Nestin antibody, (b) aNSC marker Sox2 antibody, (c) nuclei DAPI staining, and (d) merged images. Both Nestin and Sox2 showed positive staining, confirming the identity of aNSCs from *in vivo*. Scale bar, 50 µm. n=3.
(e-h) Representative immunostaining (IF) images of differentiated cells from WT aNSCs with (e) neuronal marker Tuj1 antibody, (f) glial cell marker GFAP antibody, (g) nuclei DAPI staining, and (h) merged images. Both Tuj1 and GFAP showed positive staining, confirming the pluripotency of aNSCs from *in vivo* and neural fate upon differentiation. Scale bar, 50 µm. n=3.
(i-l) Representative immunostaining (IF) images of differentiated cells from WT aNSCs with (i) aNSC marker Nestin antibody, (f) glial cell marker GFAP antibody, (g) nuclei DAPI staining, and (h) merged images. These data suggested that aNSCs underwent the differentiation with very few Nestin+ cells remaining, and produced both GFAP+ astrocytes and Tuj1+ neurons.

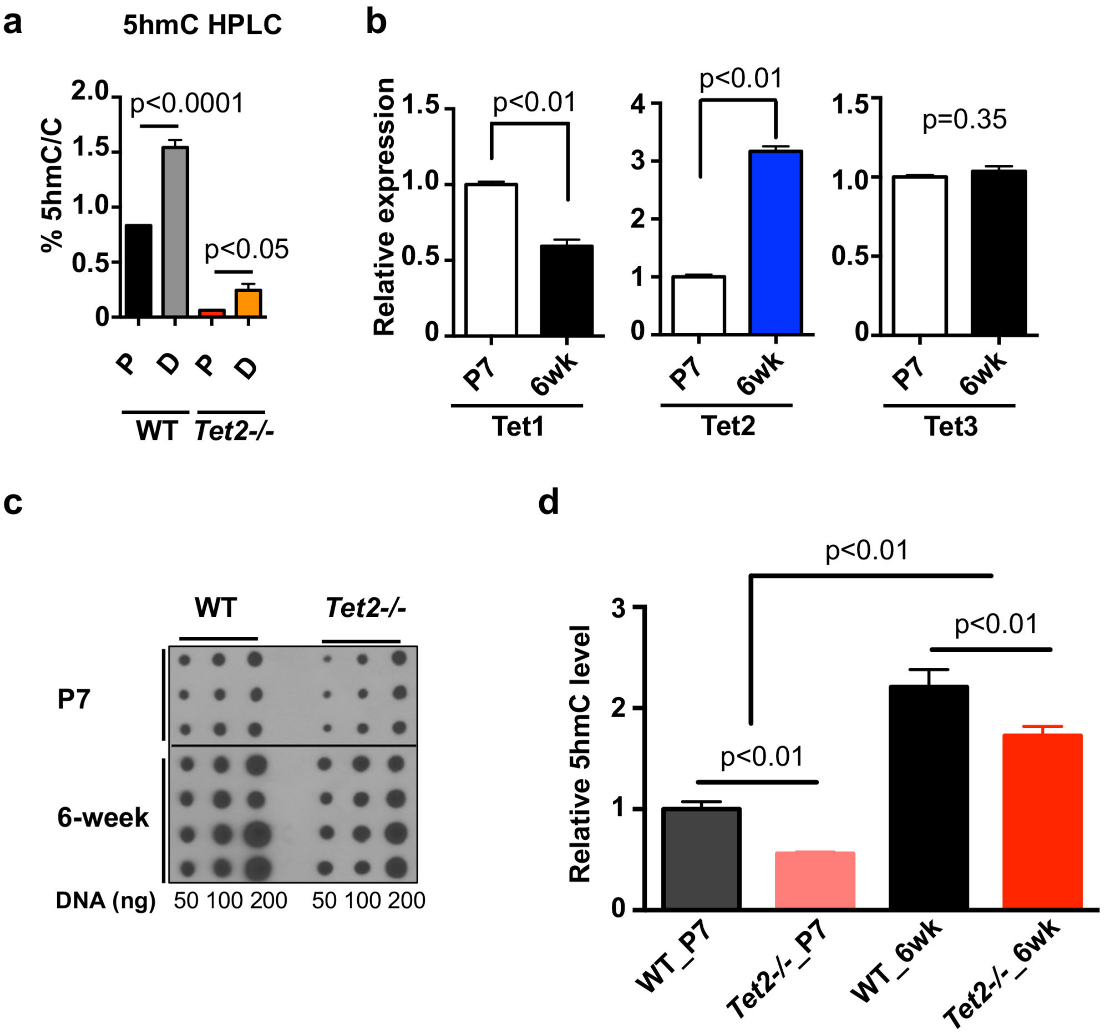

Supplementary Figure 2. Tet2 was primarily responsible for 5hmC dynamics during neurodevelopment and neurogenesis.

(a) Highly sensitive mass spectrometry (liquid chromatography–mass spectrometry (LC-MS/MS)) was applied to confirm the 5hmC dynamics between P (Proliferating) and D (Differentiated) aNSCs from both WT and *Tet2-/-* mice. Differentiation led to significant increase of 5hmC (WT-D versus WT-P, n=3; unpaired *t*-test, p<0.0001). Depletion of Tet2 resulted in a significant decrease of 5hmC in both WT and *Tet2-/-* aNSCs (n=3; unpaired *t*-test, p-values were indicated).

(b) Quantitative reverse-transcription PCR determined Tet1, Tet2, and Tet3 expression in the hippocampus of postnatal day 7 (P7) and 6-week-old (6wk) mice. Tet2 displayed a specific elevation during neurodevelopment, whereas Tet1 and Tet3 were downregulated. All data are presented as mean±SEM. (n=3; unpaired *t*-test, p-values were indicated.).

(c-d) Representative dot-blot experiments using 5hmC-specific antibody indicated a relative increase of 5hmC levels during neurodevelopment, and Tet2 depletion resulted in a significant decrease of global 5hmC level in both P7 and adult (8-10 weeks) cerebellum compared to WT. 5hmC dot-blot intensities were quantified by ImageJ. (n=3; unpaired *t*-test, p-values were indicated).

a

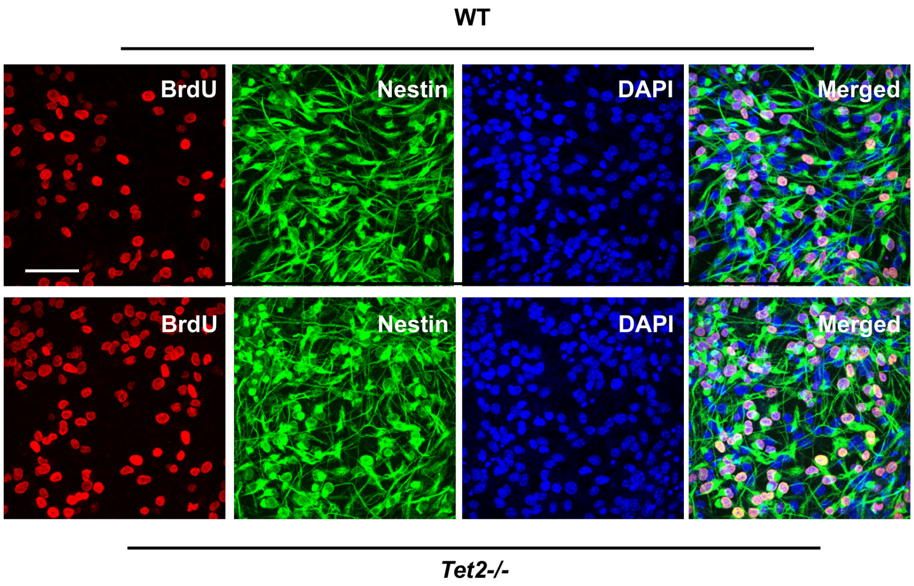

b

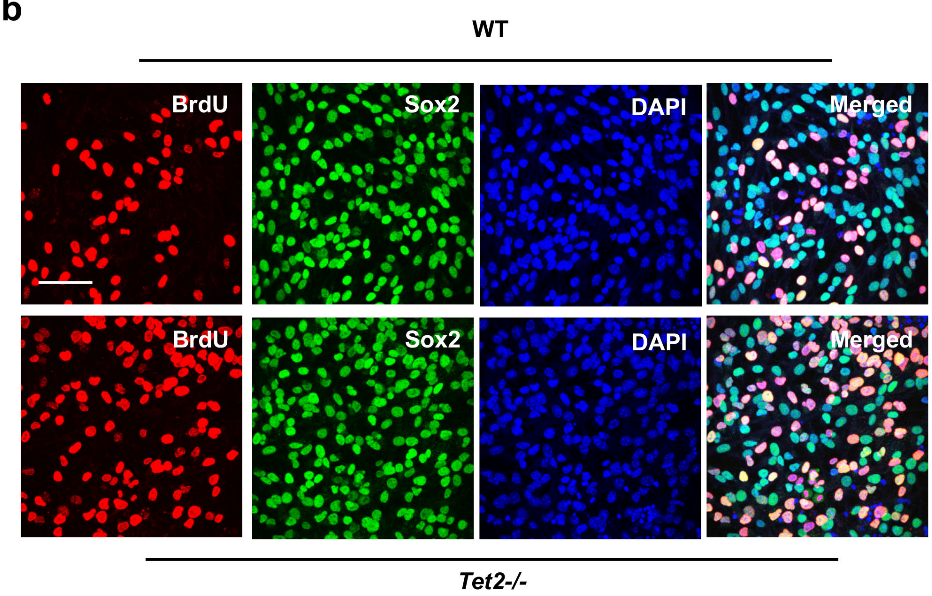

Supplementary Figure 3. Tet2 deletion led to enhanced proliferation in aNSCs.
(a) Representative immunostaining (IF) images in both WT and *Tet2-/-* aNSCs stained by proliferation marker BrdU antibody, aNSC marker Nestin antibody, nuclei DAPI staining, and merged images. Depletion of Tet2 resulted in a significant increase of aNSC proliferation (n=3; unpaired *t*-test, p<0.05).
(b) Representative immunostaining (IF) images in both WT and *Tet2-/-* aNSCs stained by proliferation marker BrdU antibody, aNSC marker Sox2 antibody, nuclei DAPI staining, and merged images. Depletion of Tet2 resulted in a significant increase of aNSC proliferation (n=3; unpaired *t*-test, p<0.05).

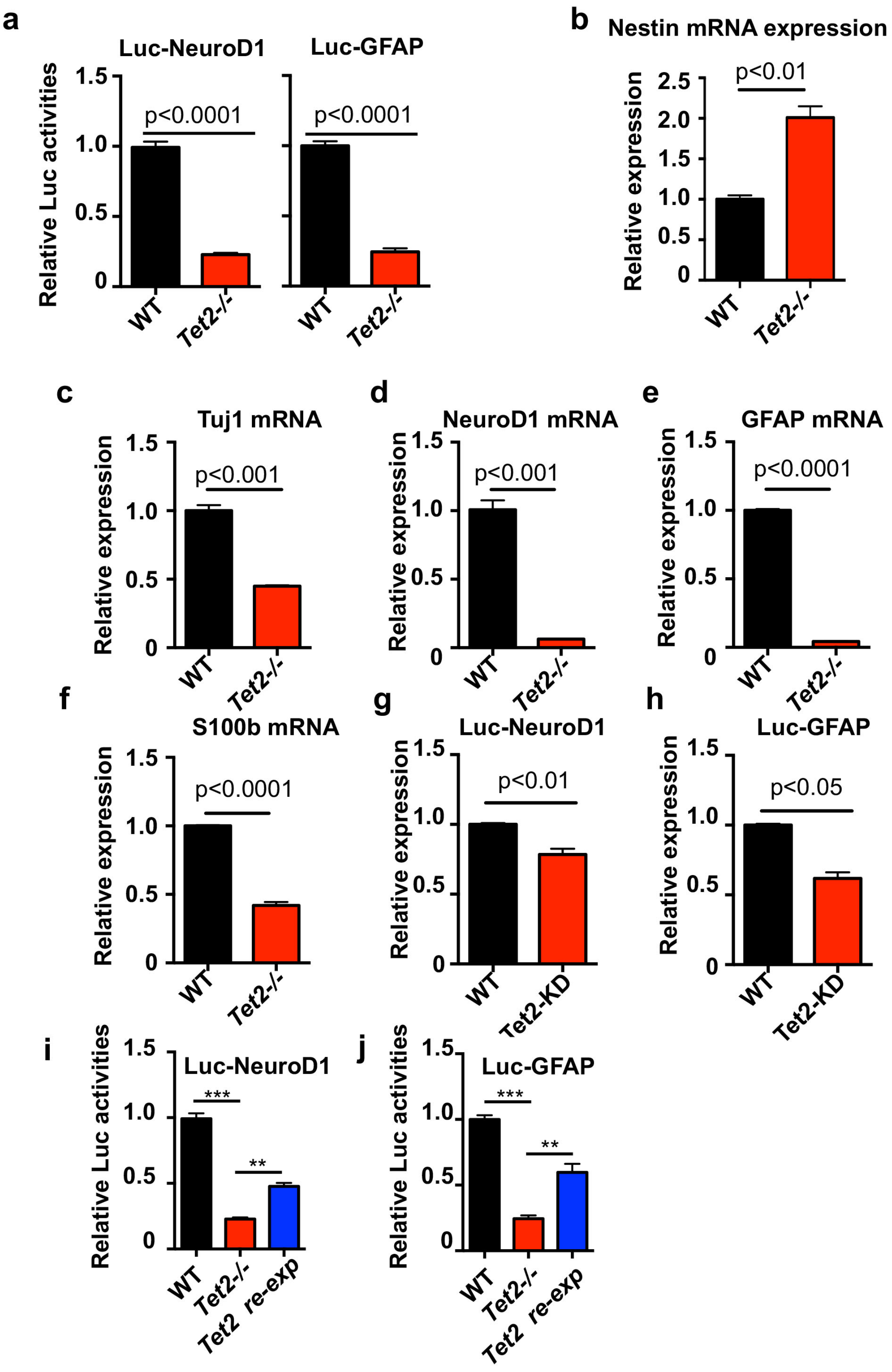

Supplementary Figure 4. Depletion of Tet2 in aNSCs led to differential expression of aNSC and neuronal/glial cell markers.

(a) Promoters of neuronal marker NeuroD1 and glial cell marker GFAP were fused with luciferase reporter to determine the epigenetic roles of Tet2 in the aNSC differentiation process by modulating these gene expressions. Relative luciferase activities were measured by a dual luciferase system. Tet2 depletion significantly reduced both luciferase reporter activities, confirming the central roles of Tet2 during this process (n=6; unpaired *t*-test, p-values were indicated.).

(b) Quantitative reverse-transcription PCR determined the aNSC marker Nestin gene expression (mRNA levels) aNSCs. Significant increases of Nestin expression were found in *Tet2-/-* aNSCs. (n=3; unpaired *t*-test, p-values were indicated.).

(c-d) Quantitative reverse transcription PCR determined the neuronal markers Tuj1 and NeuroD1 gene expression (mRNA levels) in WT and *Tet2-/-* aNSCs. Significant decreases of these neuronal markers were found in the absence of Tet2. (n=3; unpaired t-test, p-values were indicated.).

(e-f) Quantitative reverse-transcription PCR determined the glial markers GFAP and S100β gene expression (mRNA levels) in adult WT and *Tet2-/-* aNSCs. A significant decrease of these glial markers was found in the absence of Tet2. (n=3; unpaired t-test, p-values were indicated.).

(g-h) Tet2 siRNA knockdown significantly reduced both NeuroD1 and GFAP luciferase reporter activities, supporting *bona fide* regulatory roles of Tet2 on these promoters (n=3, unpaired *t*-test, p-values were indicated).

(i-j) Re-expression of Tet2 in the *Tet2-/-* aNSCs restored the NeuroD1 and GFAP reporter expression. The re-expression of Tet2 significantly restored the NeuroD1 and GFAP reporter expression comparing to *Tet2-/-* aNSCs, supporting the key roles of Tet2 in adult neurogenesis. n=3, unpaired *t*-test. **: $p<0.01$; ***: $p<0.001$.

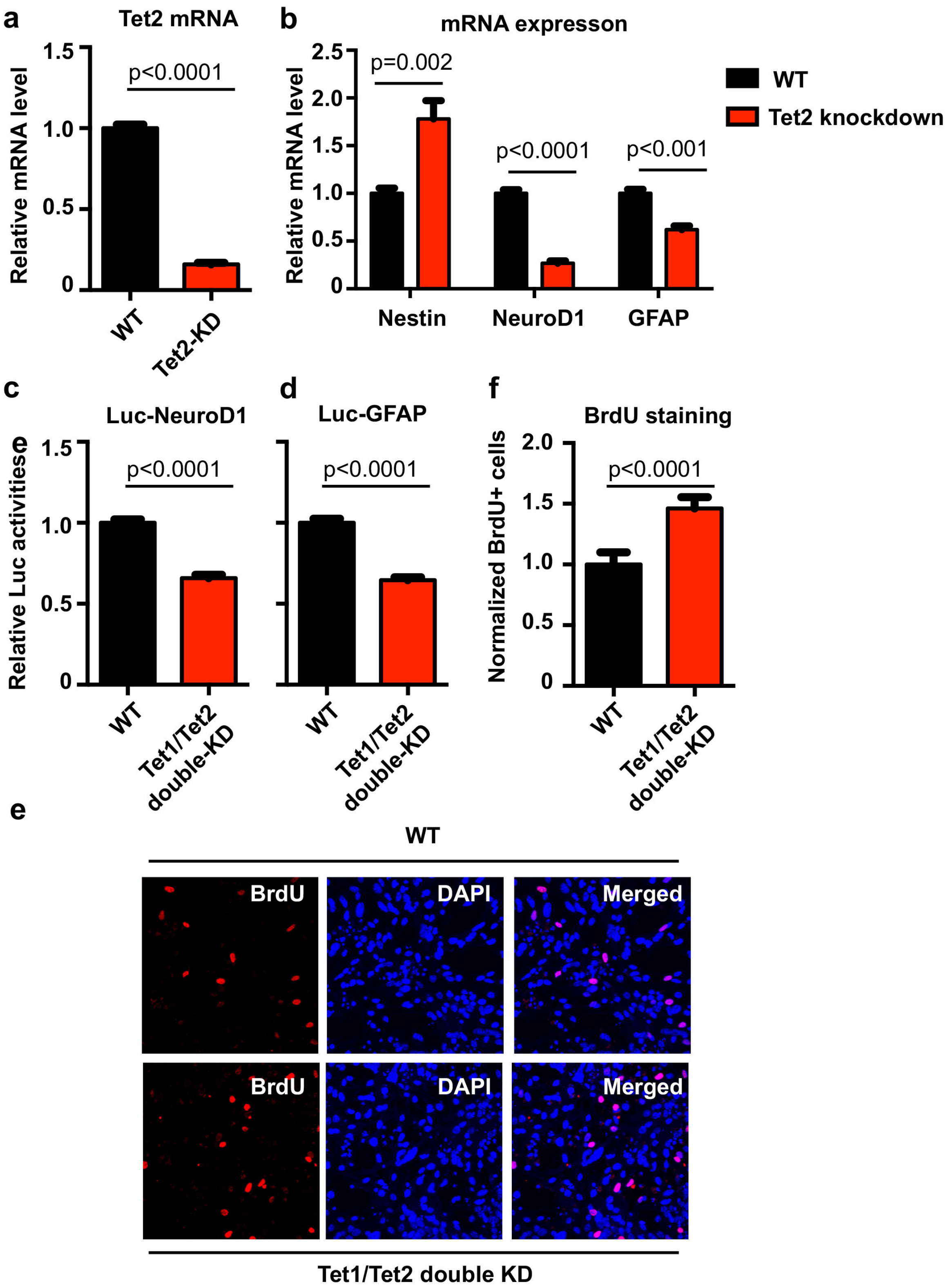

Supplementary Figure 5. Tet2 played predominant roles in aNSC proliferation and differentiation.

(a) qPCR demonstrated the effective knockdown (KD) of Tet2 by shRNA in WT aNSCs. (n=3, unpaired *t*-test, p<0.0001).
(b) Transient knockdown of Tet2 led to the significant upregulation of Nestin and downregulation of NeuroD1 and GFAP expression determined by qPCR, consistent with the enhanced proliferation and impaired differentiation in *Tet2-/-* aNSCs. (n=3; unpaired *t*-test, p-values were indicated).
(c-d) Simultaneous knockdown of both Tet1 andTet2 by shRNA led to the significant downregulation of NeuroD1 and GFAP luciferase reporters' expression, indicating the double-knockdown impaired aNSC differentiation. (n=3; unpaired *t*-test, p-values were indicated).
(e-f) Simultaneous knockdown of both Tet1 andTet2 by shRNA led to the significant increase of BrdU+ aNSCs, indicating the double-knockdown enhanced aNSC proliferation. (n=3; unpaired *t*-test, p<0.0001).

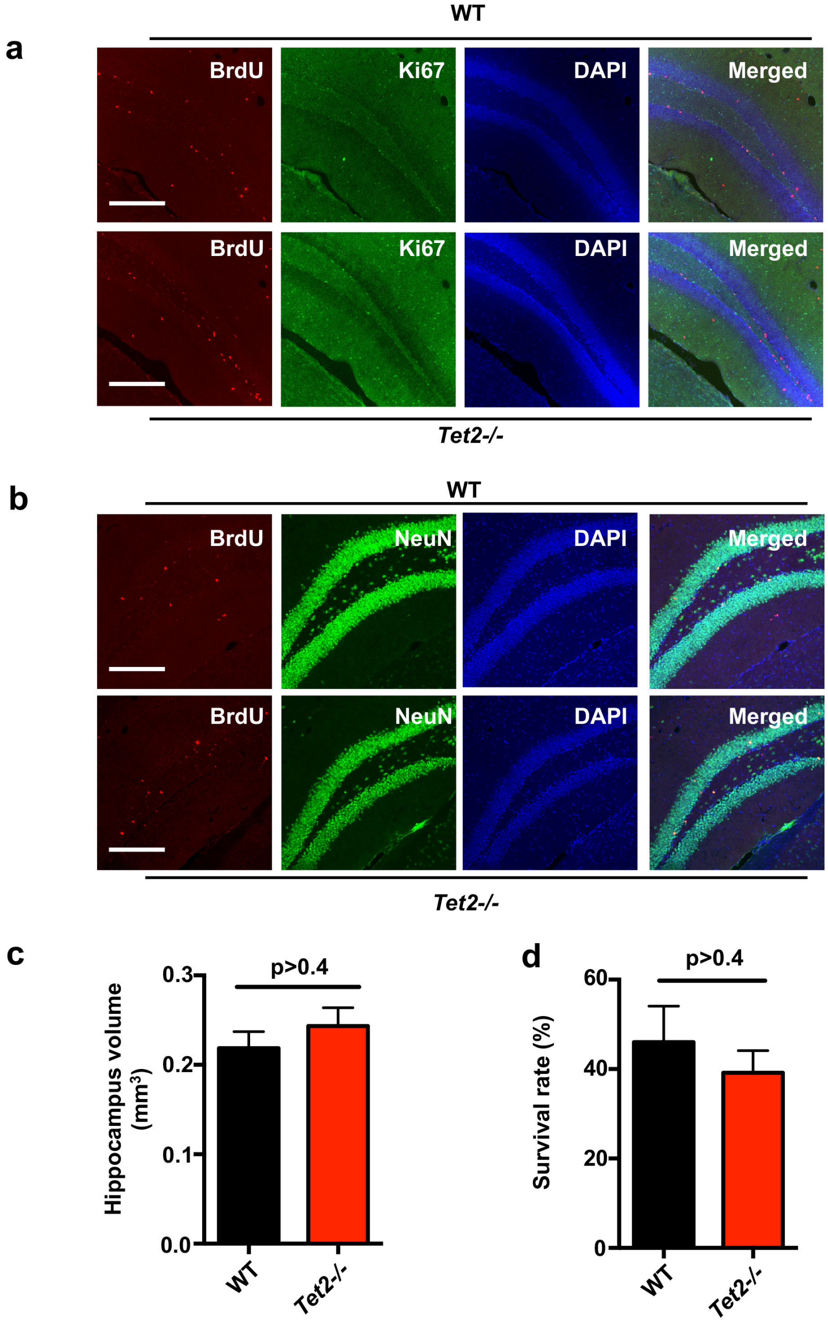

Supplementary Figure 6. Tet2 depletion led to enhanced hippocampal aNSC proliferation and impaired differentiation *in vivo*.

(a) Representative immunostaining (IF) images in the hippocampi of WT and *Tet2-/-* mice sacrificed 7 days after final BrdU administration. The hippocampi were stained by proliferation marker BrdU antibody, proliferation marker Ki67 antibody, nuclei DAPI staining, and merged images. Depletion of Tet2 resulted in a significant increase of aNSC proliferation *in vivo* (n=3; unpaired *t*-test, $p<0.05$).

(b) Representative immunostaining (IF) images in the hippocampi of WT and *Tet2-/-* mice sacrificed 4 weeks after final BrdU administration. The hippocampi were stained by proliferation marker BrdU antibody, Neuronal marker NeuN antibody, nuclei DAPI staining, and merged images. Depletion of Tet2 resulted in a significant decrease of newborn neurons marked by NeuN *in vivo* (n=3; unpaired *t*-test, $p<0.05$).
(c) Quantification of hippocampus volume indicated no difference between WT and *Tet2-/-* mice (WT, n=4; *Tet2-/-*, n=3; unpaired *t*-test, p-values were indicated.).
(d) No significant difference of the survival rates in newly generated BrdU+ cells was found between WT and *Tet2-/-* mice. (n=4; unpaired *t*-test, p-values were indicated.).

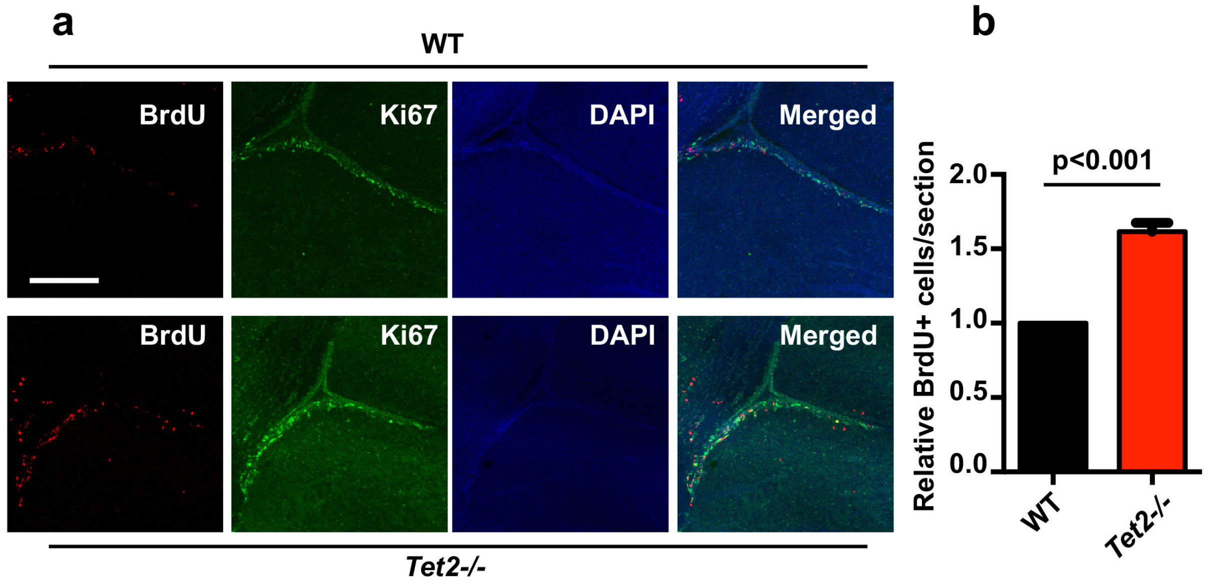

Supplementary Figure 7. Tet2 depletion led to enhanced SVZ aNSC proliferation *in vivo*.
(a-b) Representative immunostaining (IF) images in the subventricular zone (SVZ) of WT and *Tet2-/-* mice sacrificed 7 days after final BrdU administration. The SVZs were stained by proliferation marker BrdU antibody, proliferation marker Ki67 antibody, nuclei DAPI staining, and merged images. Depletion of Tet2 resulted in a significant increase of SVZ aNSC proliferation *in vivo* (n=3; unpaired *t*-test, p<0.0001).

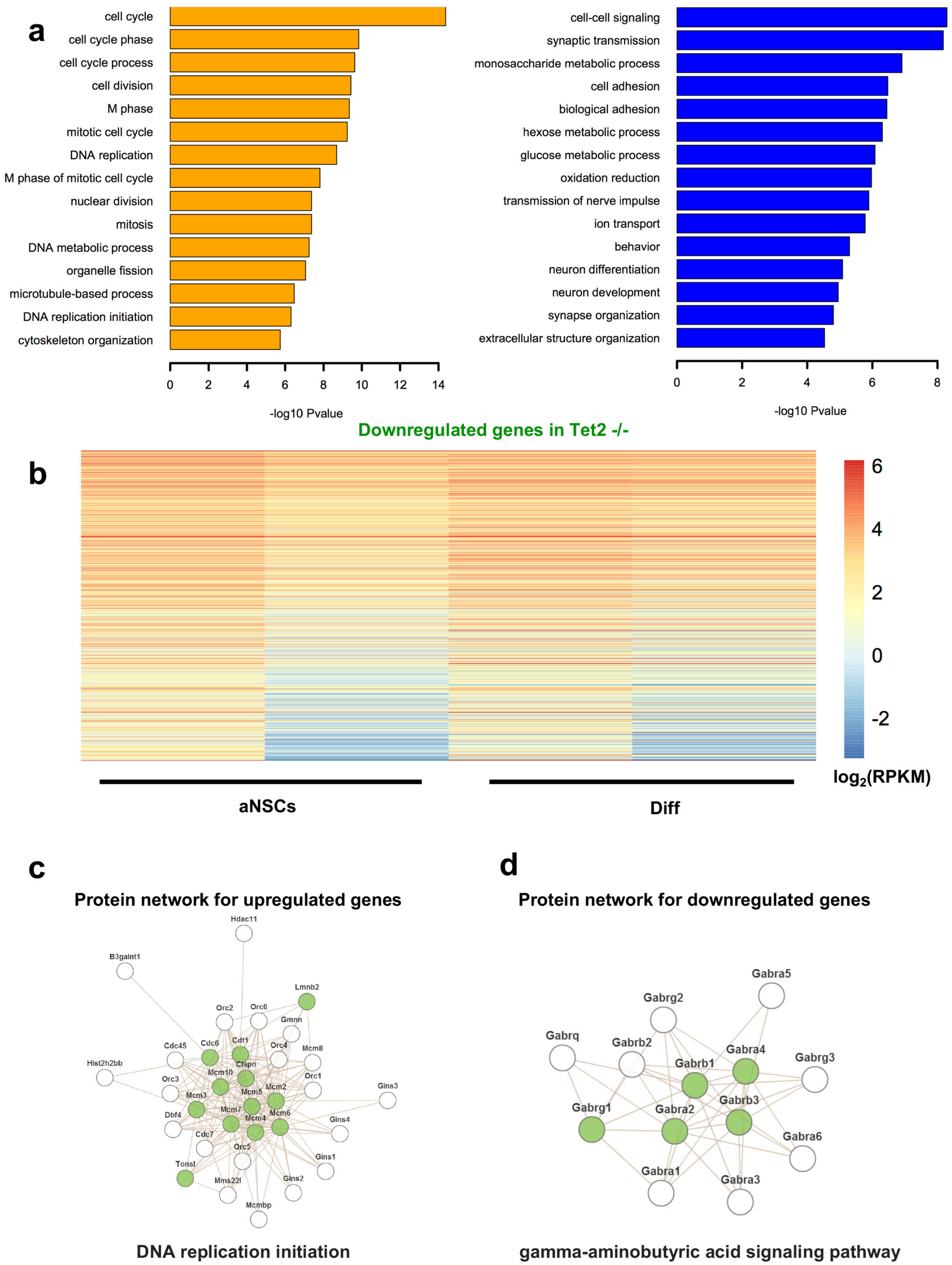

Supplementary Figure 8. Significantly altered transcriptome in the *Tet2-/-* aNSCs was consistent with enhanced aNSC proliferation and impaired differentiation.

(a) Gene ontology (GO) analysis was performed using DAVID (the database for annotation, visualization and integrated discovery) bioinformatics resources[80] using significantly up- and downregulated genes in *Tet2-/-* aNSCs. Most significant GO pathways (top Negative log10 p-values) are indicated in the bar graph. Significantly upregulated genes were enriched in GO terms involved in the cell cycle and DNA replication pathways, whereas downregulated genes participated in GO terms related to neuronal functions.
(b) Log scale of FPKM values of 979 significantly downregulated genes from WT and *Tet2-/-* proliferating aNSCs (NSCs) and differentiated aNSCs (Diff) demonstrated by heatmap view. These genes displayed similar trends in both aNSCs and Diff cells.
(c-d) Significantly up- and downregulated genes in the absence of Tet2 were further subjected to protein interaction network module analysis by the Web-based Gene Set Analysis Toolkit (WebGestalt)[82]. Networks of several genes involved in either the DNA replication initiation or gamma-aminobutyric acid signaling pathway are shown.

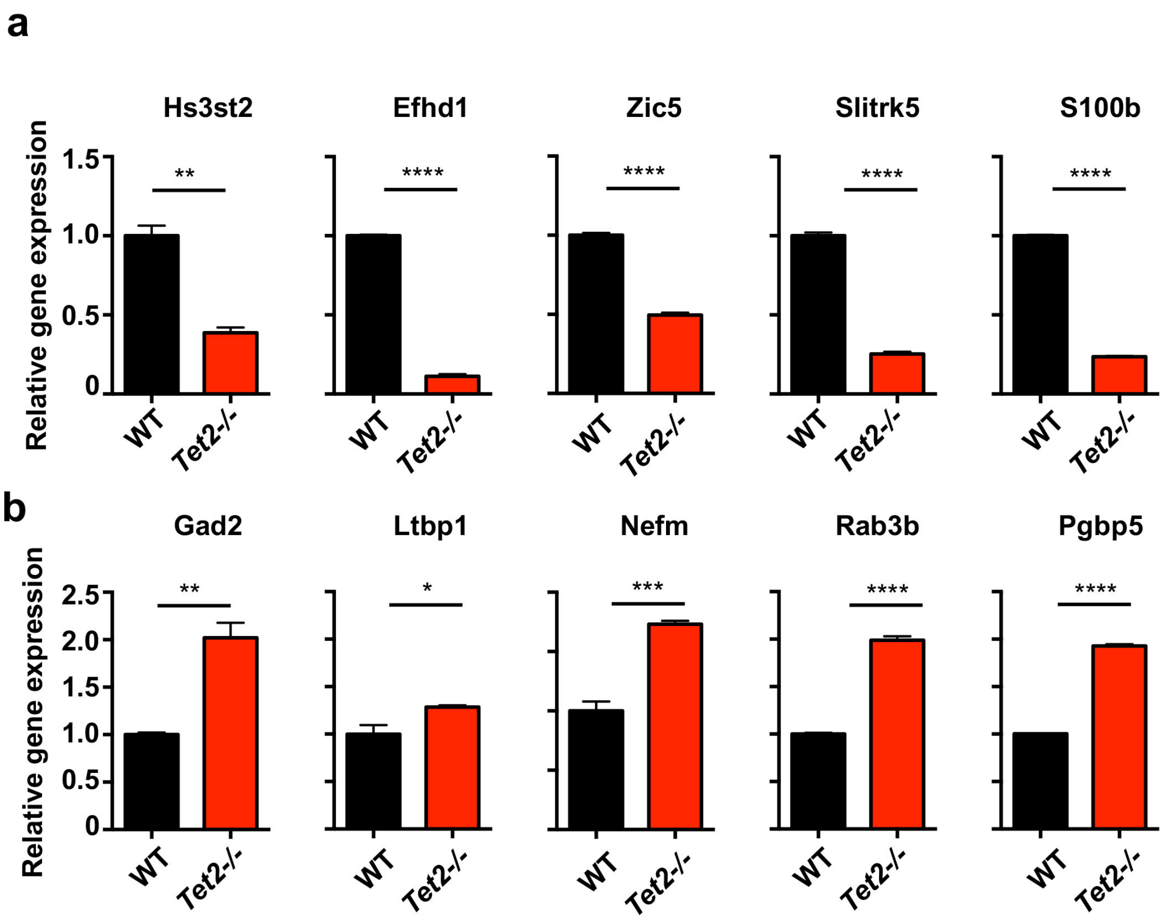

Supplementary Figure 9. Tet2 depletion led to differential expression of genes involved in aNSC proliferation and differentiation.
(a-b) Ten genes involved in aNSC differentiation (a) and cell growth/proliferation (b) were further tested by qPCR. Genes involved in aNSC differentiation were significantly downregulated, and genes involved in aNSC proliferation were significantly upregulated, which was consistent with RNA-seq results. n=3; unpaired *t*-test. *,: p<0.05; **: p<0.01; ***: p<0.001; ****:p<0.0001.

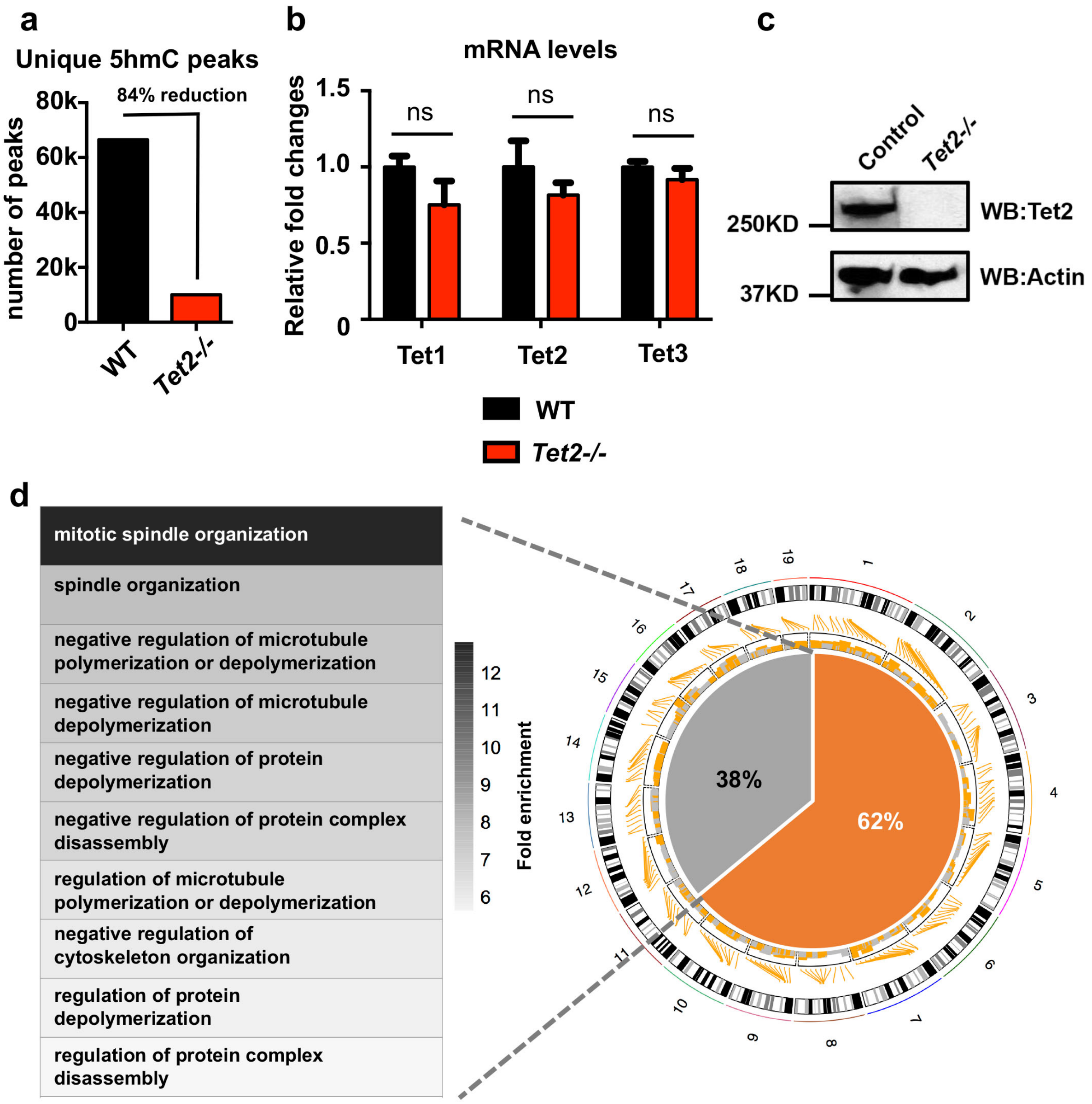

Supplementary Figure 10. Significantly upregulated genes bearing less intragenic 5hmC in the absence of Tet2 are largely enriched in protein depolymerization pathways.

(a) *Tet2-/-* aNSCs possessed 84% fewer unique 5hmC peaks compared to WT aNSCs, suggesting Tet2 is primarily responsible for 5hmC dynamics in aNSCs.

(b) The expressions of Tet protein in WT and *Tet2-/-* aNSCs were evaluated by RNA-seq. Since *Tet2-/-* mice were generated by replacing part of exon 3 sequences of the Tet2 gene with nlacZ/nGFP[41], the RNA-seq reads of Tet2 only showed marginal changes. Endogenous Tet1 and Tet3 in *Tet2-/-* aNSCs did not show significant changes, indicating the 5hmC dynamics during adult neurogenesis were primarily controlled by Tet2. (n=2, unpaired *t*-test, p-values were indicated.)

(c) Endogenous Tet2 expression at protein levels were examined by immunoblotting in both WT and *Tet2-/-* aNSCs using Tet2-specific antibody. The results demonstrated the complete depletion of Tet2 protein. Actin served as internal control.

(**d**) Circular map view of upregulated genes in the absence of Tet2 and their correlation with 5hmC are indicated. Each mouse chromosome is shown in the black-and-white outer track. Orange lines attached to the chromosomes indicate chromosomal locations of 703 upregulated genes. Intragenic 5hmC reads ratio between WT and *Tet2-/-* aNSCs were calculated on these genes and indicated in the inner track, with red bars demonstrating higher 5hmC in *Tet2-/-* aNSCs, and grey bars showing lower 5hmC in *Tet2-/-* aNSCs. Pie chart summarizes these results and indicates 38% of upregulated genes (n=264, binomial test, p>0.1) carried lower intragenic 5hmC in *Tet2-/-* aNSCs than WT. GO analyses on 264 upregulated genes bearing lower intragenic 5hmC and promoter Foxo3a in *Tet2-/-* revealed genes largely enriched in GO terms related to depolymerization. Fold enrichment is demonstrated by heatmap view.

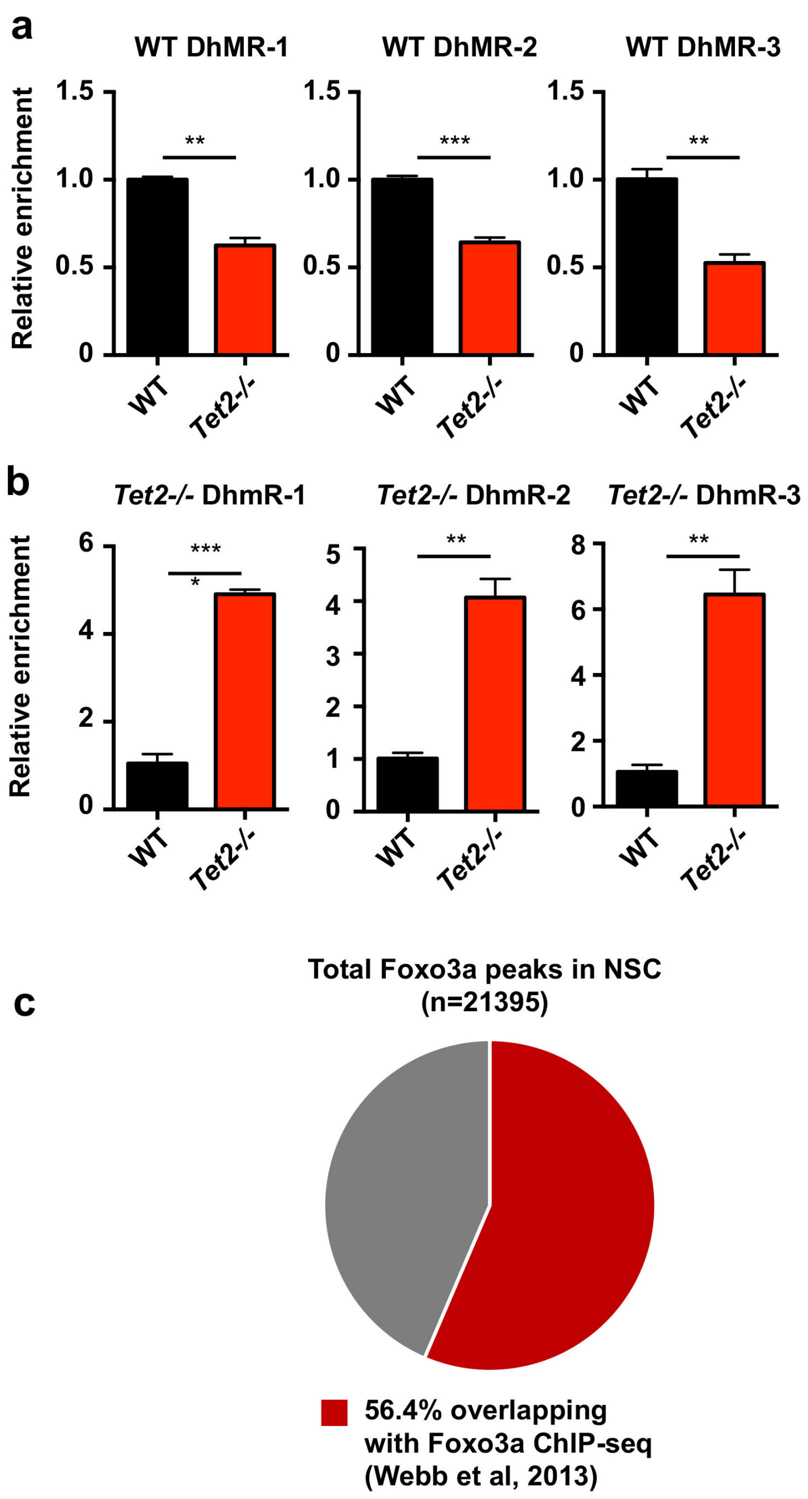

Supplementary Figure 11. Significantly upregulated genes bearing less intragenic 5hmC in the absence of Tet2 are largely enriched in protein depolymerization pathways.

(a-b) Validation of 5hmC capture-seq (hME-Seal) by qPCR. Six primer sets were designed to target 3 WT DhMRs (The levels of 5hmC in WT were higher than in *Tet2-/-* aNSC identified by hME-Seal) and 3 *Tet2-/-* DhMRs. As expected, all three WT DhMRs displayed significant higher

5hmC levels in WT than *Tet2-/-* aNSC (a), and all three *Tet2-/-* DhMRs showed significantly higher 5hmC levels in *Tet2-/-* aNSC (b). n=3; unpaired *t*-test. **: $p<0.01$; ***: $p<0.001$.
(c) Merged Foxo3a ChIP-seq peaks from WT and Tet2-/- aNSCs were used to calculate their overlap with published Foxo3a ChIP-seq peaks in cortical NSCs. 56.4% of our Foxo3a ChIP-seq peaks overlapped with published dataset.

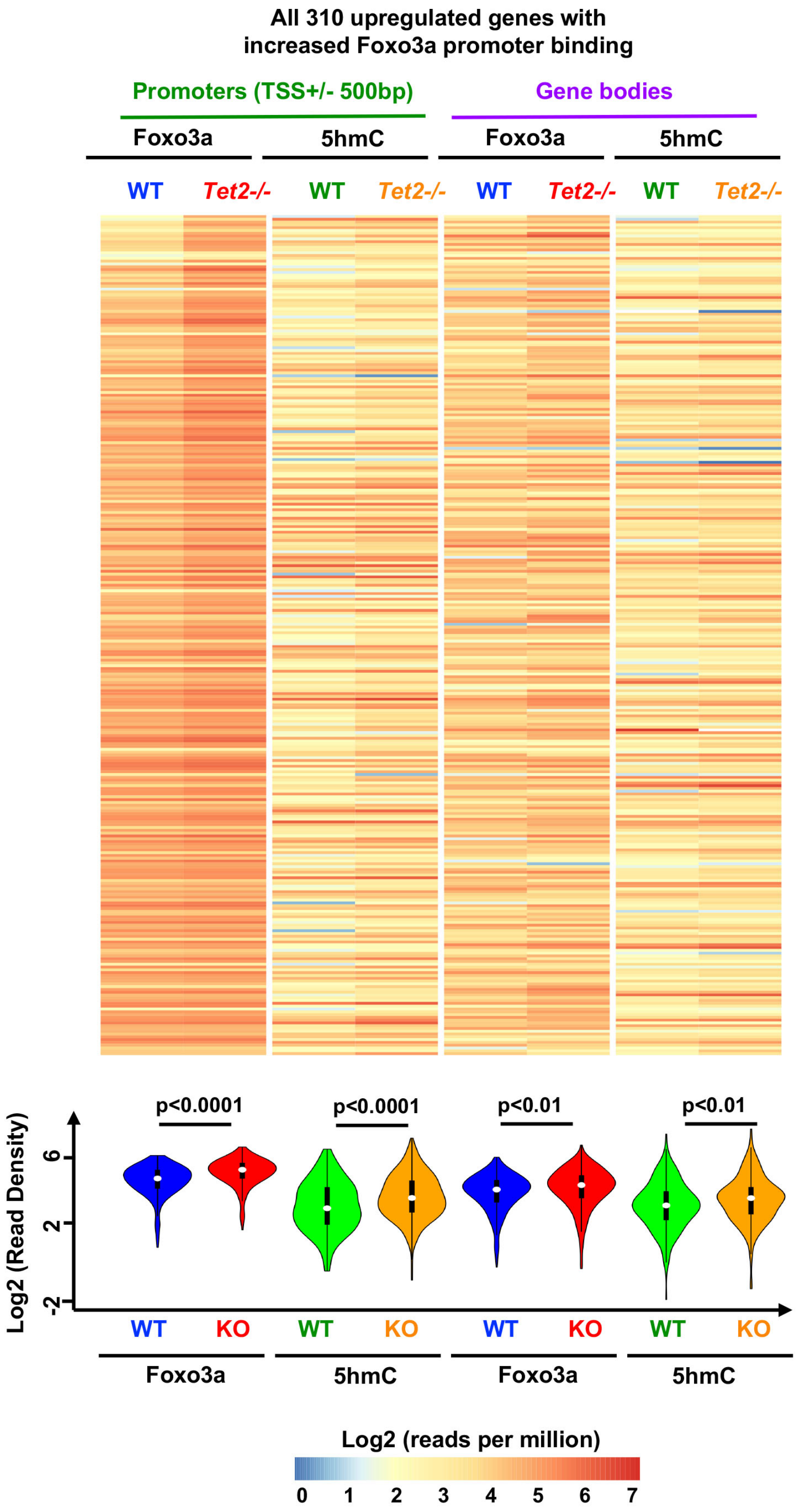

Supplementary Figure 12. Overall Foxo3a and 5hmC reads on 310 upregulated genes with increased Foxo3a promoter binding.

Average normalized Foxo3a and 5hmC mapped reads from both WT and *Tet2-/-* aNSCs were calculated on promoters (500bp upstream and downstream of transcription start sites, TSS) and

gene bodies (regions between 500bp downstream of TSS to transcription ending sites, TES) of upregulated genes with increased intragenic 5hmC and promoter Foxo3a (n=310). Log2 of average reads per million on each gene are displayed by heatmap. Violin plots summarized the mean value of each column. Statistical significance was assessed by unpaired *t*-test and p-values were indicated. Foxo3a was substantially enriched on promoters and positively correlated with 5hmC dynamics as well as transcriptional upregulation.

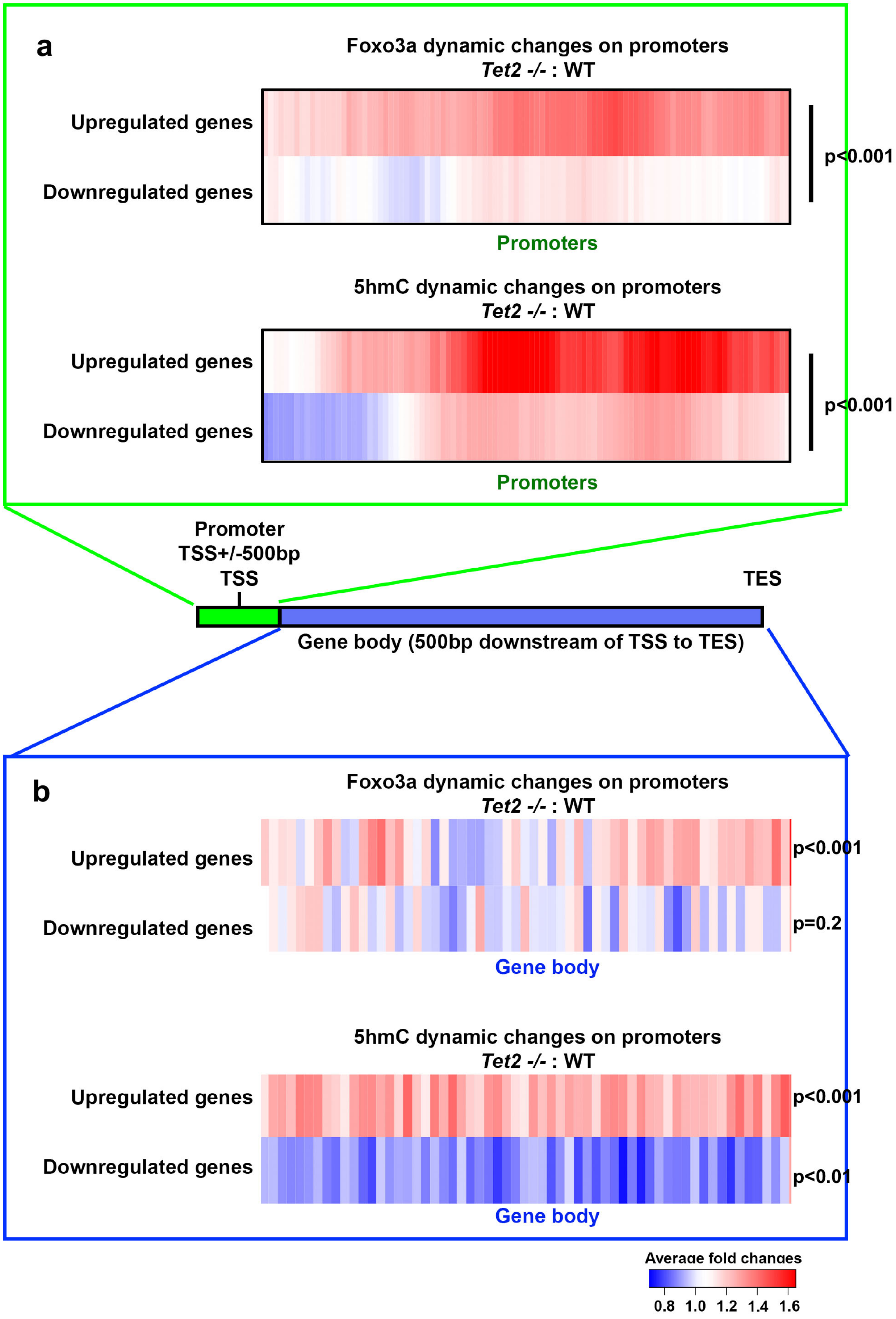

Supplementary Figure 13. Foxo3a predominantly increased on the promoters of upregulated genes in the absence of Tet2.

(a) Average fold change of Foxo3a and 5hmC normalized reads between *Tet2-/-* and WT aNSCs were calculated in the promoter regions of both upregulated and downregulated genes. The promoters were divided into 100 bins, and the ratio between normalized reads were calculated. Average fold change was plotted in Heatmap view. p-values were calculated by unpaired t-tests. Foxo3a and 5hmC showed substantial and significant increase in the promoters of upregulated genes than downregulated genes.
(b) Average fold change of Foxo3a and 5hmC normalized reads between *Tet2-/-* and WT were calculated in the gene body regions of both upregulated and downregulated genes. The gene body were divided into 100 bins, and the ratio between normalized reads were calculated. Average fold change was plotted in Heatmap view. p-values were calculated by unpaired t-tests. Foxo3a and 5hmC showed positive correlation with gene expression. No significant changes were found in Foxo3a binding on the downregulated genes. p-values were indicated.

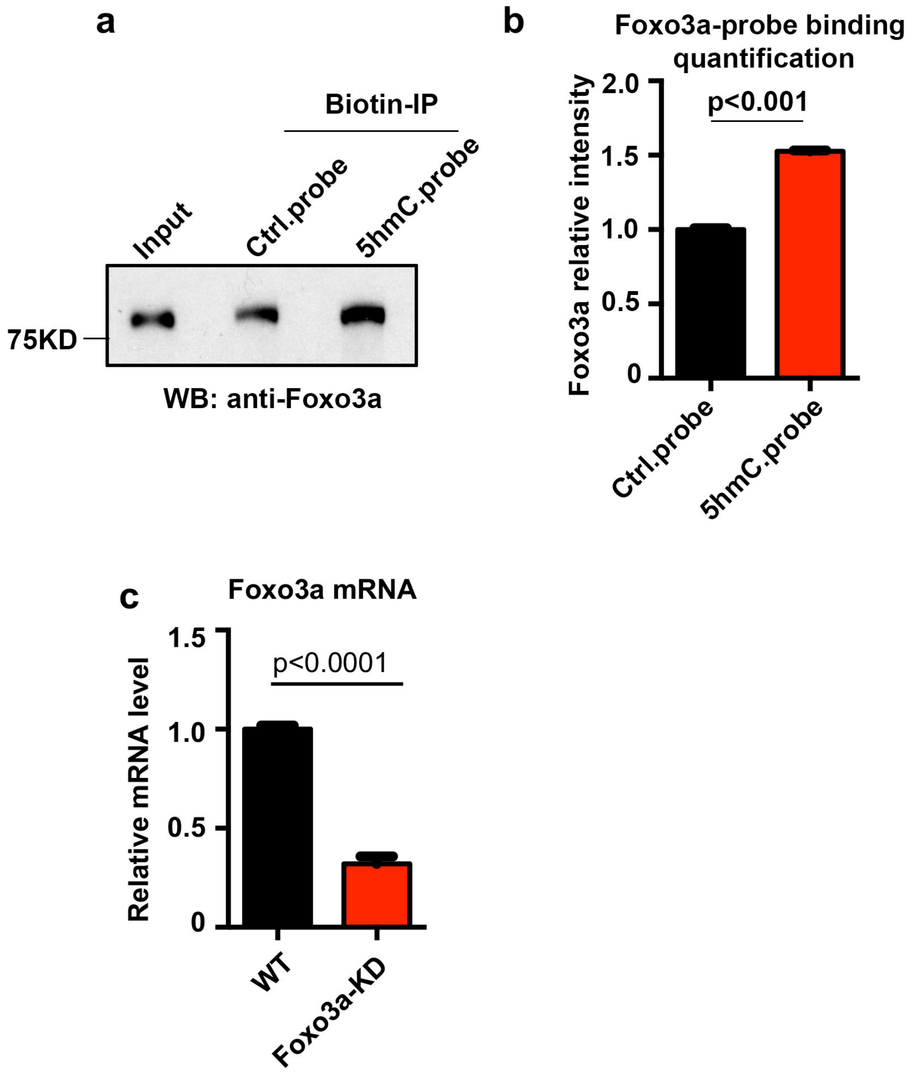

Supplementary Figure 14. Foxo3a preferentially bound to 5hmC-modified DNA consensus sequence *in vitro*.

(a) *In vitro* DNA probe-Foxo3a binding assays were performed to confirm direct and preferential interactions between 5hmC-modified DNA and Foxo3a. Both unmodified and 5hmC-modified DNA oligos with Foxo3a binding consensus identified by ChIP-seq were mixed with 1μM recombinant FOXO3A protein. Foxo3a showed a stronger affinity for the 5hmC-modified DNA oligos than unmodified oligos.

(b) Quantification of Foxo3a-probe binding indicated a significant higher affinity of FOXO3A to 5hmC modified probe. (n=3; unpaired *t*-test, p<0.001).

(c) qPCR demonstrated the effective knockdown (KD) of Foxo3a by shRNA in *Tet2-/-* aNSCs. (n=3, unpaired *t*-test, p<0.0001).

Supplementary Figure 15

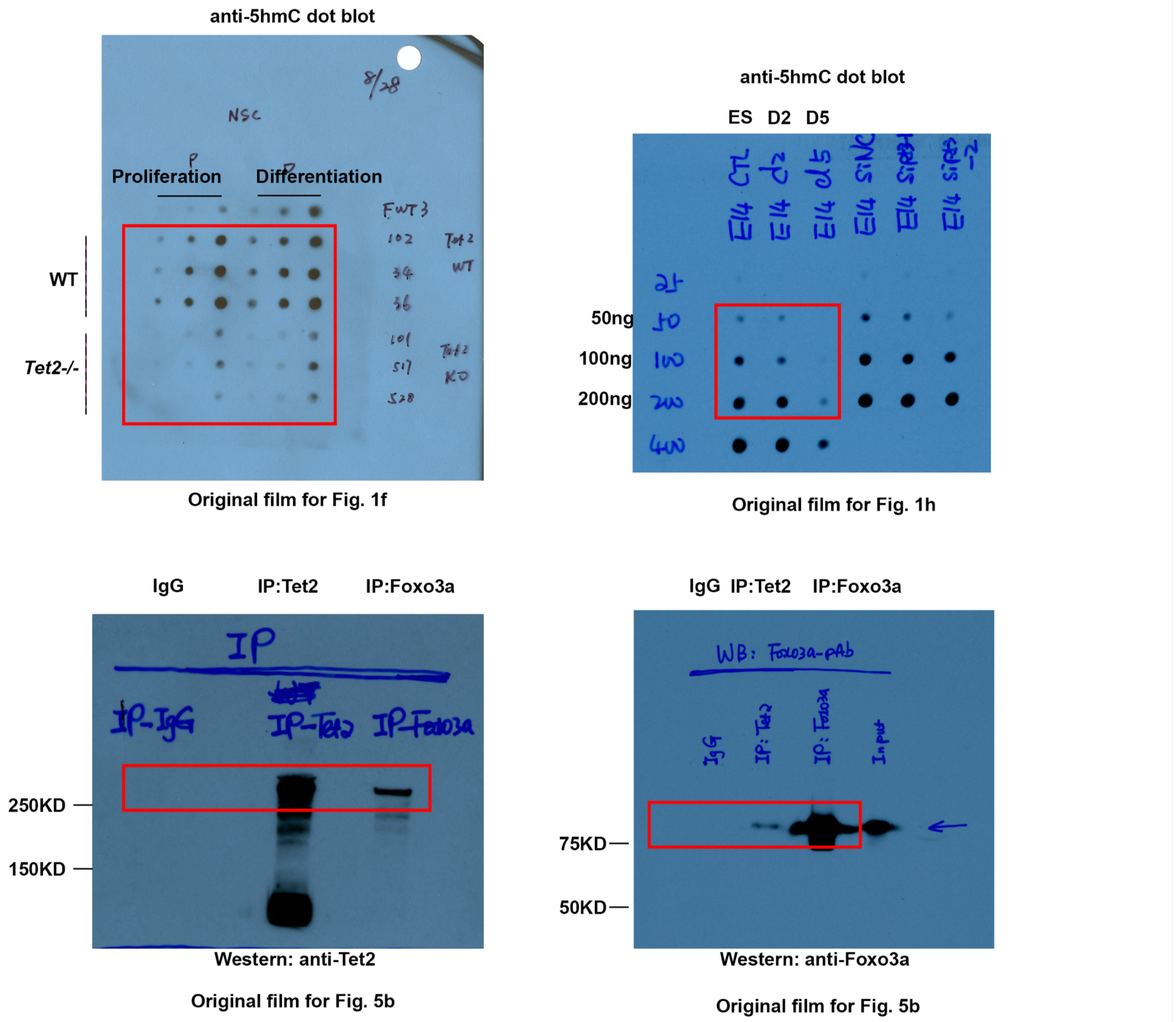

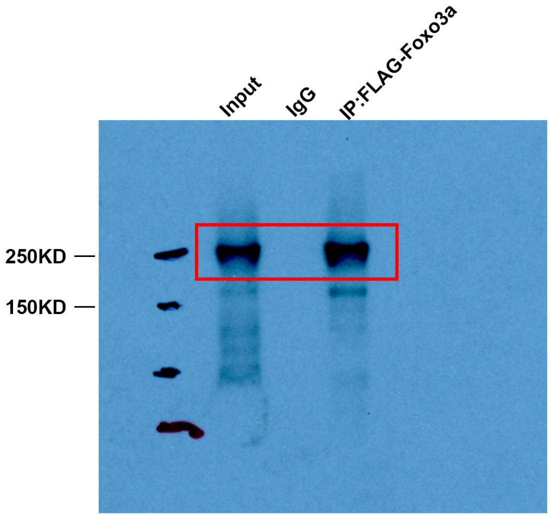

Western: anti-Myc (Tet2)

Original film for Fig. 5c

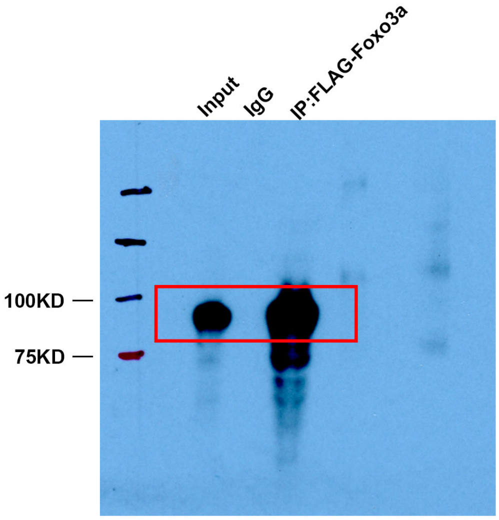

Western: anti-FLAG (Foxo3a)

Original film for Fig. 5c

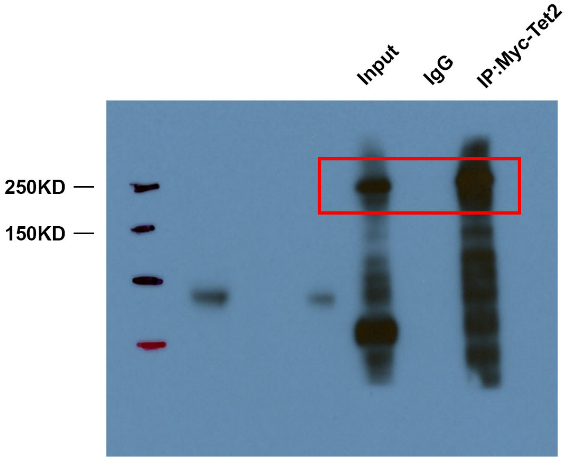

Western: anti-Myc (Tet2)

Original film for Fig. 5c

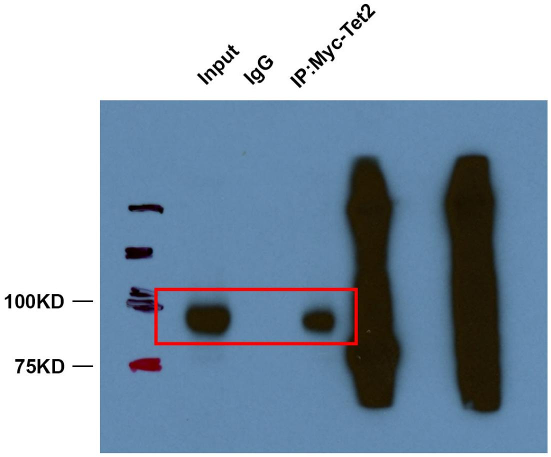

Western: anti-FLAG (Foxo3a)

Original film for Fig. 5c

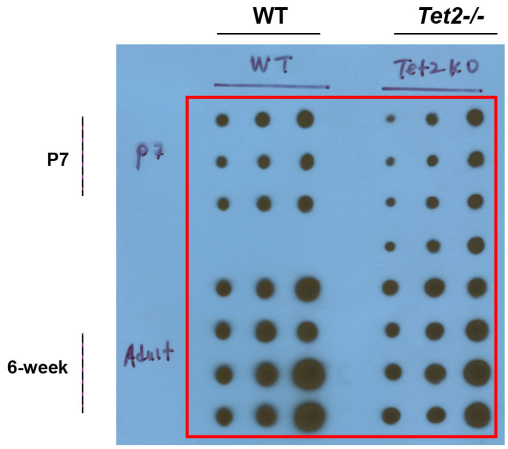

Original film for Supplementary Figure 2c

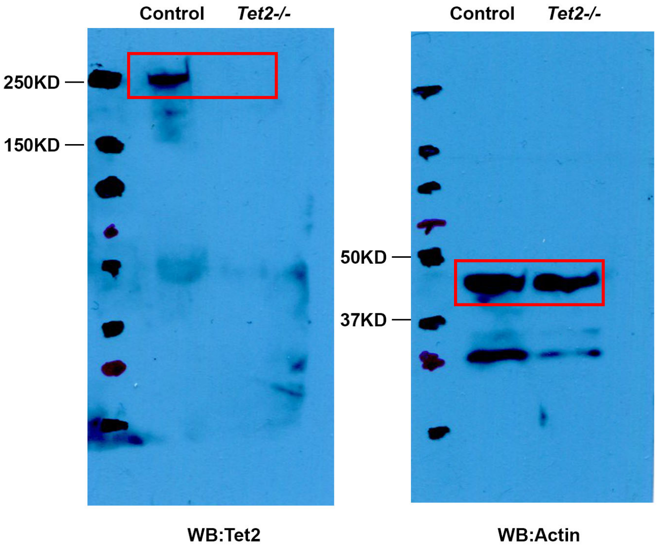

Original film for Supplementary Figure 10c

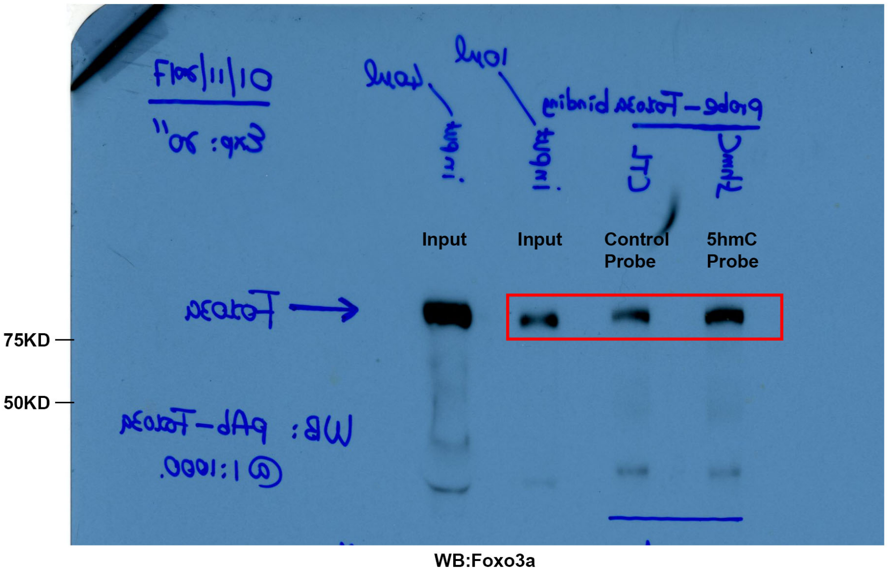

Original film for Supplementary Figure 14a

Supplementary Figure 15. Original films for dot blots and western blots in figures. Original films for dot blots and western blots used in the main and supplementary figures as indicated. The cropped area of each figure was highlighted by red box.
